# Age- and sex-specific incidence rates and future projections for hip fractures in Zimbabwe

**DOI:** 10.1136/bmjgh-2024-017365

**Published:** 2025-01-27

**Authors:** Hannah Wilson, Tadios Manyanga, Anya Burton, Prudance Mushayavanhu, Joseph Chipanga, Samuel Hawley, Kate A Ward, Simon Graham, James Masters, Tsitsi Bandason, Matthew L Costa, Munyaradzi Ndekwere, Rashida A Ferrand, Celia L Gregson

**Affiliations:** 1 University of Bristol Musculoskeletal Research Unit, Bristol, Bristol, UK; 2 The Health Research Unit Zimbabwe at the Biomedical Research and Training Institute, Harare, Zimbabwe; 3 Department of Surgery, Sally Mugabe Central Hospital, Harare, Zimbabwe; 4 Department of Surgery, Midlands State University, Gweru, Zimbabwe; 5 MRC Lifecourse Epidemiology Centre, Human Development and Health, Southampton, UK; 6 MRC Unit The Gambia at London School of Hygiene and Tropical Medicine, Banjul, Gambia; 7 Oxford Trauma and Emergency Care, Nuffield Department of Orthopaedics, Rheumatology and Musculoskeletal Science, University of Oxford, Oxford, UK; 8 Clinical Research Department, London School of Hygiene & Tropical Medicine, London, UK

**Keywords:** Global Health, Epidemiology, Public Health, Cohort study

## Abstract

**ABSTRACT:**

**Introduction:**

Population ageing in Africa is increasing healthcare demands. Hip fractures require multidisciplinary care and are considered an indicator condition for age-related health services. We aimed to estimate current hip fracture incidence in Zimbabwe, compare rates against other regional estimates and estimate future fracture numbers.

**Methods:**

All hip fracture cases in adults aged ≥40 years, presenting to any hospital in Harare over 2 years, were identified. From this, age- and sex-specific hip fracture incidence rates per 100 000 person-years were estimated using 2022 Zimbabwean Census data and compared with South African and Botswanan estimates. Furthermore, using the United Nations population projections, future hip fracture numbers were estimated to 2052 for Zimbabwe.

**Results:**

In 2022, 1 83 312 women and 1 79 212 men aged ≥40 years were living in Harare (14.9% of the city’s population). Over 2 years 243 hip fracture cases, 133 (54.7%) female, mean (SD) age 71.2 (15.9) years, were identified. Most presented to public hospitals (202 [83.1%]) and were fragility hip fractures (211 [86.8%]); high-impact trauma (eg, traffic accidents) was more common in younger men. Presentation delays of >2 weeks were common (37.4%). Incidence rates for adults aged ≥40 years in Harare (observed) and Zimbabwe (estimated) were 33.5 and 53.8/100 000 person-years, respectively. Over age 50, rates increased with age, with the highest rates seen in women aged ≥85 years (704/100 000 person-years). Age-standardised hip fracture incidence rates are broadly comparable between Zimbabwe, Botswana and Black South Africans in those aged 40–69 years; thereafter, rates in Zimbabwean women and men exceed those in Botswana and South Africa. Across Zimbabwe, the number of hip fractures occurring annually is expected to increase more than 2.5-fold from 1709 in 2022 to 4414 by 2052.

**Conclusion:**

In Zimbabwe, most hip fractures in adults ≥50 years are fragility fractures, consistent with age-associated osteoporosis; incidence rates exceed those previously reported regionally. Demands on already challenged healthcare systems will increase.

**WHAT IS ALREADY KNOWN ON THIS TOPIC**

- Across Africa, life expectancy is increasing, and age-associated diseases are placing new demands on already overstretched healthcare services. Hip fractures require complex multidisciplinary care and are considered an indicator condition for age-related health services.
- Other than in South Africa, hip fracture incidence has not been prospectively studied in West, East or Southern Africa. Only a retrospective study had been undertaken in Botswana.

**WHAT THIS STUDY ADDS**

- Age-standardised hip fracture incidence for men and women aged ≥40 years in Zimbabwe was 46.6 and 58.8 per 100 000 person-years, respectively.
- In older adults, hip fracture incidence rates in Zimbabwean women and men exceed those in Botswana and South Africa.
- Most hip fractures in those aged ≥50 years are fragility fractures, consistent with age-associated osteoporosis.
- Across Zimbabwe, the number of hip fractures occurring annually is expected to increase more than 2.5-fold in the next 30 years.

**HOW THIS STUDY MIGHT AFFECT RESEARCH, PRACTICE OR POLICY**

- While most fractures in Africa stem from high-energy trauma, for example, road traffic accidents, we have shown that in the case of hip fractures in adults aged ≥50 years, most are fragility fractures indicative of osteoporosis. As age increases, fragility fractures increase.
- Currently no anti-osteoporosis medicines are featured in the WHO Essential Medicines list; evidence of osteoporotic fracture burdens in ageing African populations should influence change.
- With these data, fracture risk assessment tools, such as FRAX can now be calibrated for routine use in clinical practice in Zimbabwe.
- Hip fracture projections are intended to inform national planning of orthopaedic services.

## Introduction

Life expectancy is increasing in many African populations.^1^ By 2050, the number of people aged over 60 years is expected to reach 156 million in West, East and Southern Africa.^2^ Ageing increases the risk of many comorbidities, including musculoskeletal diseases, exemplified by osteoporotic fragility fractures of the hip, often attributable to low levels of trauma such as falling from standing.^3^ Across Africa, health systems that have hitherto largely focused on maternal and child health and infectious diseases^4^ are now also needing to pivot to address the demands of age-related diseases.

Hip fractures have a major impact on a person’s health and quality of life^5^ and on healthcare services as they require complex multidisciplinary care. In Europe, osteoporotic fragility fractures account for a remarkable 3.5% of all healthcare spending, of which hip fractures are a substantial contributor.^6^ The Global Burden of Disease study determined that in 2019 there were 178 million new fracture cases worldwide (including high- and low-energy trauma fractures), an increase of 33.4% since 1990.^7^ Notably, and contrary to historic perceptions, bone density is similar in Black African and White European populations,^8^ suggesting that we can expect to see an increasing burden of age-associated osteoporotic fractures in Africa, as has been seen in Europe over recent decades.^9^ Ageing populations in the African region experience specific contextual challenges potentially influencing the fracture risk, for example, additional burdens of malnutrition,^10^ high HIV prevalence^11^ and high trauma rates.^12^ In Zimbabwe (population 15.1 million),^13^ an estimated 42% of the population was living in poverty in 2022,^14^ 12.9% of adults live with HIV^15^ and there are 1721 road trauma deaths a year.^16^

To date, only two studies have quantified hip fracture incidence in the West, East or Southern African population. In Botswana, a retrospective review of 435 hip fracture cases from 2009 to 2011 suggested that hip fracture incidence was low^17^, while another larger prospective study of 2767 hip fractures in South Africa (2017 to 2018) showed that although hip fracture incidence rates were lowest in Black South Africans compared with other ethnic groups, the absolute number was greatest given 81% of the population are Black South Africans.^18 19^ Understanding the current and future country-specific hip fracture incidence rates can inform both health service planning and individual fracture risk assessment because incidence estimates can be used to calibrate commonly used clinical fracture risk assessment tools, such as FRAX.^20^ This assessment tool is currently available in over 80 countries, but in West, East or Southern Africa, only South Africa and Botswana have an FRAX tool calibrated using country-specific data.^21^

We aimed to determine current age- and sex-specific hip fracture incidence rates in older adults in Harare Province, Zimbabwe, and use these data to first compare against other regional hip fracture incidence rates and second to estimate the number of future hip fractures for the country.

## Methods

### Study design and setting

A prospective cohort study was conducted to determine hip fracture incidence. In Zimbabwe, our study area was the capital province, Harare, an urban/peri-urban area with a population of 2.4 million people, reflecting 15% of the population.^13^ Eight hospitals provide all hip fracture care in the city: two government-funded public hospitals and six private hospitals. Community cases can present to these hospitals directly or to one of the 42 polyclinics in Harare Province. Primary healthcare clinics (known as polyclinics in Zimbabwe) are staffed by nurses and refer to the two public hospitals. Both public hospitals, all polyclinics and five private hospitals agreed to permit data collection on site. One small private hospital that very rarely saw a hip fracture declined. A community-based network was established, and every polyclinic and all seven hospitals were sensitised to hip fracture identification, in discussion with facility managers and healthcare staff. Posters were displayed, and WhatsApp groups were established to aid timely identification of cases. Hospitals were visited or contacted daily and polyclinics were at least weekly to monitor new potential hip fracture presentations.

### Study population

Adults aged 40 years or older, residents in Harare Province, who had sustained a hip fracture and were presenting for the first time to any of the seven hospitals or 42 polyclinics were eligible to be included. As some adults have both an urban and a rural home, residency was defined as it is in the Zimbabwean national census^13^—by where the person stayed the night before their injury.

### Data collection

Data were collected prospectively for all incident hip fracture cases presenting over 2 years (12 October 2021 to 12 October 2023). Data were collected by a trained researcher and entered directly into pre-programmed Research Electronic Data Capture (REDCap) questionnaires (hosted by the University of Bristol) with inbuilt data validation, working offline on Samsung Galaxy tablets. REDCap is a secure, web-based software platform designed to support data capture for research studies.^22 23^ Data included age, sex, region of residence, presentation date, time since injury (dichotomised as within the last 2 weeks or more than 2 weeks ago), hip fracture classification (based on radiographic diagnosis) and the trauma mechanism (high-energy trauma, eg, road traffic accident or low-energy trauma, eg, fall from a standing height).

### Hip fracture classification

Hip fractures were confirmed by reading radiographs wherever possible. Each radiograph was reviewed by two orthopaedic surgeons and classified as intracapsular (International Classification of Diseases, 10th Revision [ICD-10] code S72.0), pertrochanteric (ICD-10 code S72.1) and sub-trochanteric (ICD-10 code S72.2). If a radiograph had been taken, but was not made available to the research team, the hip fracture classification was taken from the orthopaedic opinion documented in the hospital medical records. Where no radiograph (eg, due to X-ray equipment faults, interrupted electricity supply, unaffordability of radiographs), the case history was used by an orthopaedic surgeon to determine a clinical diagnosis of a hip fracture. This was defined as (i) a fall where the patient landed on their buttocks or side, after which (ii) they quickly experienced severe pain in the groin or hip with or without radiation down to the knee and (iii) a shortened and externally rotated leg was evident on examination.

### Statistical analysis

Characteristics of the hip fracture population were described using numbers with percentages, and means with SD. X^2^ tests were used to determine associations between patient characteristics and (a) delayed presentation and (b) mechanism of injury.

#### Hip fracture incidence

The number of hip fracture cases was counted over 2 years and the mean per year was calculated; 2 years of data collection increased the precision of incidence estimates. The contemporaneous Zimbabwe 2022 Population and Housing Census provided the total population of Zimbabwe, stratified by sex and 5 year age band.^13^ The Census provided the same data for urban dwelling persons (many of whom live in Harare Province; 41%). These proportions were applied to the total number of men and women living in Harare Province, to estimate the number of adults in each 5 year age and sex strata, resident in Harare Province, as these data are not provided directly by the Census.

Age- and sex-specific hip fracture incidence rates per 100 000 persons per year in Harare were calculated as the number of fractures divided by the at-risk population (adults in each age/sex strata age ≥40 years living in Harare Province in 2022). The overall incidence per 100 000 in Harare in adults aged ≥40 was calculated as the total number of fractures in 1 year divided by the at-risk population (ie, all adults aged ≥40 years living in Harare Province in 2022). Age- and sex-specific incidence rates were used to determine the relative risk ratio with 95% CI for each age band, using the 40–44 year age band as the reference group. For women, the 45–49 year age band was used as the reference group because no hip fractures were recorded in women aged 40–44 years. National hip fracture incidence rates per 100 000 persons per year in 2022 were calculated by standardising the observed Harare age- and sex-specific incidence rates to the age and sex structures in Zimbabwe in 2022 (ie, multiplied by the proportion of the Zimbabwe population in each of the sex/age strata).

To determine whether fragility fracture incidence rates were similar, a sensitivity analysis was performed excluding all hip fractures incurred following a high-energy injury, re-calculating Harare Province and Zimbabwe incidence rates.

#### Hip fracture incidence projections

Yearly population projections for Zimbabwe, for adults aged ≥40 years, by 5 year age band and sex, were obtained from the United Nations (UN) population prospects using the medium fertility estimates for 2022 to 2052.^2^ As we had previously found UN population projections to underestimate older age groups in South Africa,^24^ a comparison between the UN 2022 population prediction (based on data from the 2012 Census)^25^ against the actual 2022 Census figures showed that in all age categories 45 years and older (and both sexes), the UN predictions from 2012 had underestimated the Zimbabwean population in 2022, and in the 40–44 years category the UN predictions had overestimated the population size. Therefore, a correction factor was applied to all age and sex categories for the UN population projections from 2023 to 2052, using the proportional difference between the 2022 Census data and 2022 UN estimates.

Hip fracture incidence rates per 100 000 projected for Zimbabwe up to 2052 were then estimated, assuming the current incidence rates remained stable. This was calculated by standardising the observed Harare incidence rates to the projected age structure in Zimbabwe for each future year up to 2052. The predicted numbers of hip fractures per year were calculated by multiplying the Harare Province age- and sex-specific incidence rates by the 5 year age and sex band population projections and dividing by 100 000, for each future year through to 2052.

#### Cross-country comparison using available hip fracture incidence estimates in Southern Africa

We compared the age-standardised incidence rates between Zimbabwe and published data from a prospective study in South Africa^18^ and a retrospective study in Botswana.^17^ In Zimbabwe, the ethnicity of the population is 99.6% Black African.^13^ In Botswana, 94.7% of the reported hip fracture cases were among those who were Black African.^17^ We used the age-standardised incidence for Black South Africans rather than the whole country to make the results more comparable.^18^ The Botswana analysis^17^ presented crude hip fracture incidence, so age-standardised incidence was calculated in the same method as described above (the proportion of the population in each age band multiplied by the crude incidence).

Statistical analyses and graphs were created using R version 4.3.3 and R Studio version 2024.04.1+748.^26^

### Ethical and governance approvals

Ethical and governance approvals were obtained from The Medical Research Council of Zimbabwe (14/07/2021 ref MRCZ/A/2706), The Biomedical Research and Training Institute (19/02/2021 ref AP161/2021), Sally Mugabe Central Hospital (29/01/2021 ref HCHEC/ 250121/06), Parirenyatwa Group of Hospitals (25/02/2021), Harare City Health (27/01/2021) and The Research Council of Zimbabwe (RCZ, 14/07/2021 refs 04246 and 04248). These permissions allowed the collection of a minimum dataset on all hip fracture cases without the need for individual-level patient consent to avoid selection bias.

### Patient and public involvement

Hip fracture patients experience a lot of pain after their injury and suffer high levels of anxiety and distress. Therefore, we did not consider it appropriate to ask for their input into this observational study; however, we did engage multiple community-based and hospital-based healthcare personnel to fully understand the pathway of patient care to ensure in the design of our study that we maximised all chances of identifying every hip fracture in Harare Province.

## Results

### Characteristics of the hip fracture population

In total, 243 patients presented with hip fracture over the 2 year period: 83.1% to one of the two public facilities and 16.9% to one of five private facilities (table 1). All polyclinic presentations went on to present to a hospital. The overall mean age was 71 years (15.9), with just over half being female (54.7%). Of those hip fractures diagnosed by radiograph, the most common fracture types were intracapsular (37.0%) and pertrochanteric (31.7%), with relatively few subtrochanteric fractures (9.5%). The study orthopaedic team was able to access 179 (73.7%) radiographs to verify the ICD-10 classification. In a further 11 (4.5%), a radiograph was taken, but it was unavailable for verification; hence, the fracture classification was taken from the orthopaedic opinion documented in the hospital medical records. Over the 2 years, 53 people (21.8%) did not have a radiograph performed.

**Table 1:** Characteristics of patients presenting to hospital with a hip fracture in Harare Province, Zimbabwe, October 2021 to October 2023

	Number(Total n=243)	%
Age, mean (SD)	71.2 (15.9)	
Female sex	133	54.7
Presenting to a public hospital	202	83.1
Presentation delayed by >2 weeks from injury	91	37.4
Low-impact mechanism of injury (ie, a fragility fracture)	211	86.8
Hip fracture classification		
Intracapsular	90	37.0
Pertrochanteric	77	31.7
Subtrochanteric	23	9.5
Clinical diagnosis of a hip fracture (no radiograph)	53	21.8

Most hip fractures (86.8%) followed low-energy injuries consistent with fragility fractures (table 1). High-energy fractures (eg, from road traffic accidents) were more common in men than women (27 [24.5%] vs 5 [3.8%], p<0.01) and those younger than 50 years, compared with those ≥50 years (19 [59.4%] vs 13 [6.2%]; p<0.01). There were no high-energy fractures in men or women over the age of 70 years.

Delays in hospital presentation were common, with 37% reporting their injury occurred more than 2 weeks before they presented to the hospital. Those who were older (age ≥75 years) were equally likely to be delayed in presenting to a hospital as those younger (42 [17.3%] vs 49 [20.2%], p=0.73). Men and women were equally likely to present within 2 weeks (67 [60.9%] vs 85 [63.9%], respectively, p=0.73). High-energy fractures were no more likely than low-energy fractures to present within 2 weeks of injury (21 [65.6%] vs 131 [62.1%] respectively, p=0.85).

### Hip fracture incidence rates

**Figure 1:**
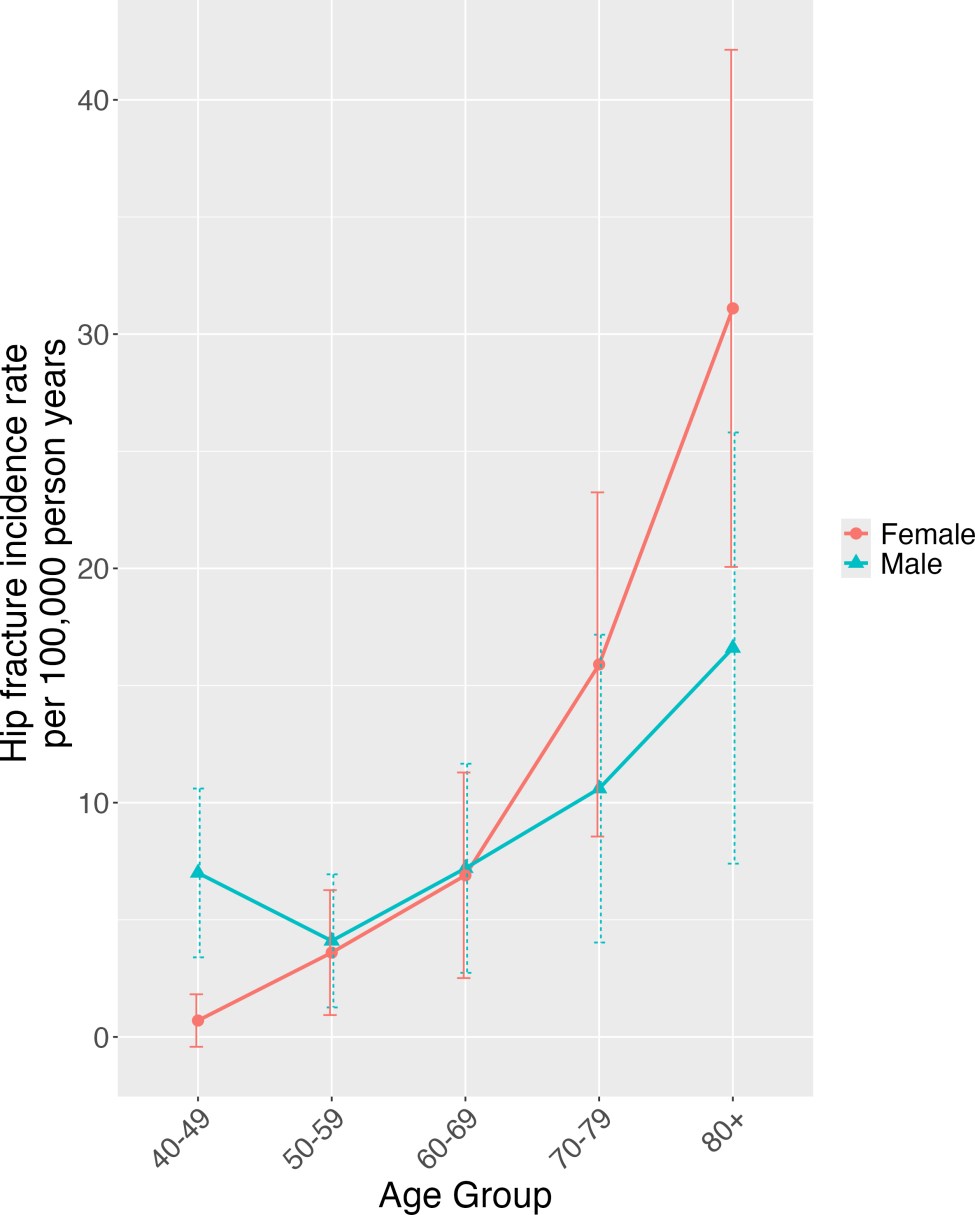
Sex-specific hip fracture incidence rates per 100 000 person-years in adults aged ≥40 years in Zimbabwe in 2022. Rates shown by 10 year age group, with 95% CIs.

In 2022, there were an estimated 362 524 adults (179 212 male, 183 312 female) aged ≥40 years living in Harare Province and 3 203 964 (1 460 544 male, 1 743 420 female) in the country of Zimbabwe. The overall incidence rates for the total population of adults aged ≥40 years in Harare Province and in Zimbabwe were 33.5 and 53.8 per 100 000 person-years, respectively (table 2). In both sexes, after the age of 50 years, there was a progressive increase in the incidence and relative risks of hip fractures with older age (figure 1, table 2). Incidence rates were markedly higher in men younger than 50 years compared with women; this appeared to be largely driven by high-energy trauma as the difference was partially attenuated after sensitivity analysis excluding high-energy traumatic injuries (onlinesupplemental table 1  figure 1). Rates were then similar in men and women until age 70 years when rates in women increased above those for men. Similarly, while the overall national incidence rate was higher in women than men (58.8 vs 46.6 per 100 000 persons per year over 40), this appeared to be driven by higher rates in women aged ≥70 years; in younger age groups, national incidence rates were higher in men than in women (figure 1, table 2).

**Table 2:** Hip fracture incidence in 5 year age bands, overall and stratified by sex, for Harare Province and Zimbabwe, 2022

Five-year age bands (years)	Number hip fractures* per year(n)	Harare province population† (n)	Incidence rate per 100 000 person years	Relative risk ratio(95% CI)	Zimbabwe total population‡(n)	Incidence rates per 100 000, age-standardised to Zimbabwe population 2022§
Men and women						
40–44	7.0	111 012	6.3	Reference	795 275	1.6
45–49	9.0	85 744	10.5	1.7 (0.63 to 4.56)	661 444	2.2
50–54	9.0	57 308	15.7	2.5 (0.93 to 6.71)	450 604	2.2
55–59	6.0	34 687	17.3	2.7 (0.91 to 8.03)	309 004	1.7
60–64	9.5	26 881	35.3	5.6 (2.11 to 14.86)	295 155	3.3
65–69	10.0	20 172	49.6	7.9 (3.01 to 20.75)	254 317	3.9
70–74	12.5	11 344	110.2	17.5 (6.94 to 44.1)	171 193	5.9
75–79	15.5	6848	226.3	35.9 (14.7 to 87.6)	110 023	7.8
80–84	14.5	4389	330.4	52.4 (21.3 to 129.1)	76 105	7.8
85+	28.5	4140	688.4	109.2 (47.8 to 249.5)	80 844	17.4
Total over 40	121.5	362 524	33.5	5.3 (2.47 to 11.4)	3 203 964	53.8
Total over 50	105.5	165 768	63.6	10.1 (4.70 to 21.7)	1 747 245	50.0
Total over 65	81.0	46 893	172.7	27.4 (12.7 to 59.3)	692 482	42.8
Men						
40–44	7.0	55 727	12.6	reference	385 120	3.3
45–49	7.5	45 200	16.6	1.3 (0.46 to 3.64)	328 502	3.7
50–54	5.0	30 666	16.3	1.3 (0.41 to 4.10)	224 187	2.5
55–59	3.0	16 308	18.4	1.5 (0.39 to 5.80)	128 893	1.6
60–64	5.0	12 063	41.4	3.3 (1.05 to 10.4)	117 633	3.3
65–69	5.0	8878	56.3	4.5 (1.43 to 14.2)	103 166	4.0
70–74	3.5	4592	76.2	6.1 (1.69 to 22.0)	67 666	3.5
75–79	6.5	2657	244.6	19.5 (6.71 to 56.7)	44 665	7.5
80–84	3.0	1680	178.6	14.2 (3.68 to 54.8)	31 067	3.8
85+	9.5	1441	659.3	52.5 (19.8 to 139.2)	29 645	13.4
Total over 40	55.0	179 212	30.7	2.4 (1.09 to 5.27)	1 460 544	46.6
Total over 50	40.5	78 285	51.7	4.1 (1.84 to 9.14)	746 922	39.6
Total over 65	27.5	19 248	142.9	11.4 (4.97 to 26.1)	276 209	32.2
Women						
40–44	0.0	55 285	0.0		410 155	0.0
45–49	1.5	40 545	3.7	Reference	332 942	0.7
50–54	4.0	26 642	15.0	4.1 (0.63 to 26.8)	226 417	1.9
55–59	3.0	18 379	16.3	4.4 (0.62 to 31.2)	180 111	1.7
60–64	4.5	14 818	30.4	8.2 (1.29 to 52.0)	177 522	3.1
65–69	5.0	11 294	44.3	12 (1.94 to 74.4)	151 151	3.8
70–74	9.0	6751	133.3	36 (6.39 to 202.7)	103 527	7.9
75–79	9.0	4191	214.7	58 (10.3 to 326.6)	65 358	8.0
80–84	11.5	2709	424.5	114.7 (20.9 to 628.5)	45 038	11.0
85+	19.0	2699	704.0	190.3 (36.1 to 1002.7)	51 199	20.7
Total over 40	66.5	183 312	36.3	9.8 (1.94 to 49.4)	1 743 420	58.8
Total over 50	65.0	87 483	74.3	20.1 (3.98 to 101.4)	1 000 323	58.1
Total over 65	53.5	27 645	193.5	52.3 (10.3 to 265.0)	416 273	51.4

* MThe mean annual number of hip fractures collected between October 2021 to-October 2023. Population of Harare province in 2022. Population of Zimbabwe in 2022. Annual incidence rate age-standardized to total Zimbabwe population over .

† Population of Harare province in 2022.

‡ Population of Zimbabwe in 2022.

§ Annual incidence rate age-standardised to total Zimbabwe population over 40 years.

### Projected hip fracture incidence rates and numbers to 2052

**Figure 2:**
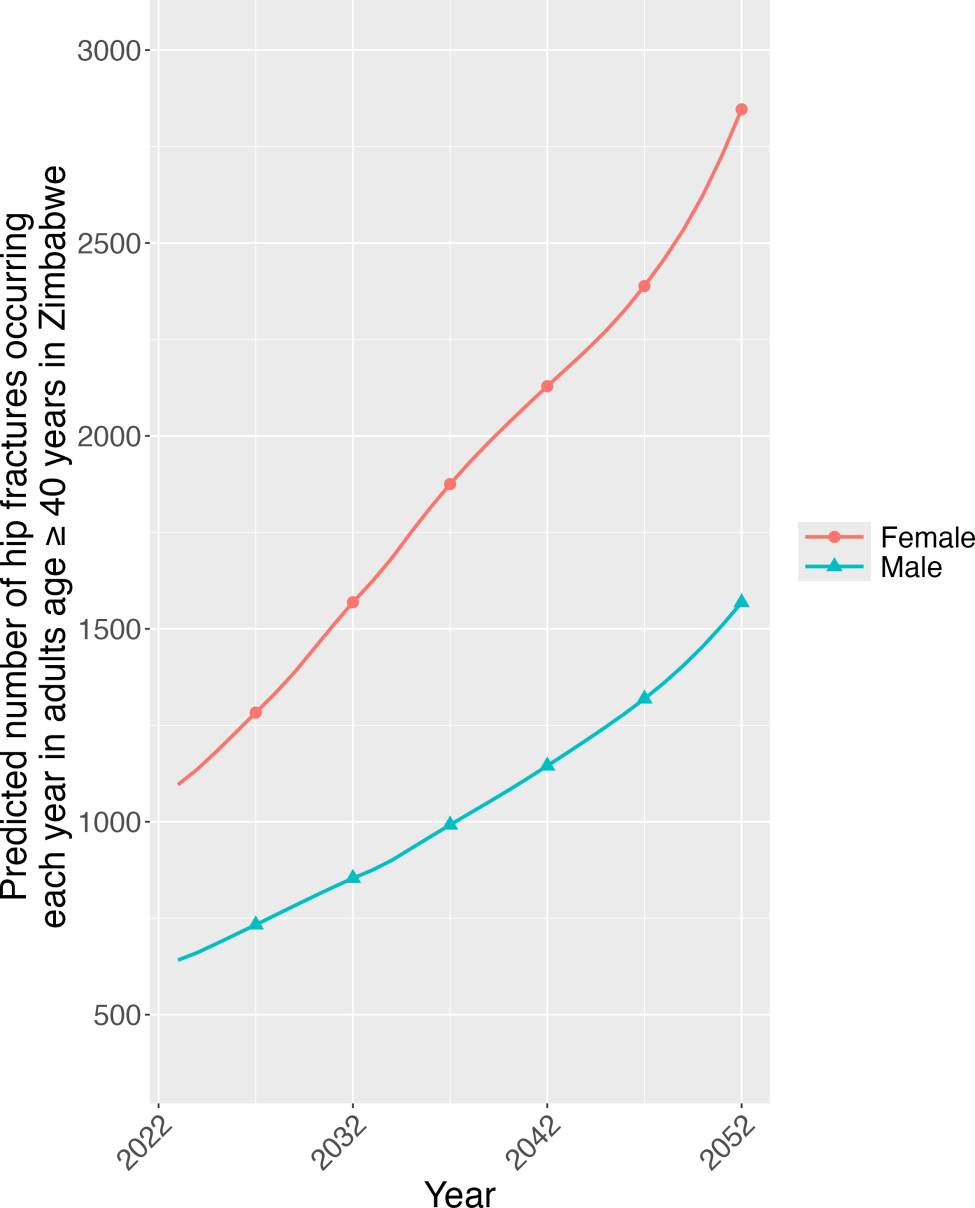
Predicted total number of hip fractures occurring in men and women each year from 2022 to 2052 in Zimbabwe.

The UN population predictions, with the correction factor applied, showed that adults aged 40 to 54 will likely experience the greatest population growth over the next 30 years (1.9 million in 2022 to 4.8 million in 2052), and the female population aged over 80 years will likely grow more than their male counterparts (increase of 80 032 women vs 20 642 men) (online supplemental figure 2). The projected age-standardised incidence in those agde ≥40 years from 2022 to 2052 is predicted to remain steady for women (58.8 to 59.1 per 100 000) while slightly reducing in men (46.6 to 41.9 per 100 000) (online supplemental figure 3). However, across Zimbabwe, the overall absolute number of hip fractures occurring each year in adults aged ≥40 years is expected to increase from 1709 in 2022 to 2423 in 2032, 3274 in 2042, to reach 4414 by 2052 (figure 2). Such that the number of hip fractures is expected to more than double in both men and women by 2052 (1027 to 2846 and 682 to 1568, respectively).

### Cross-country comparison of hip fracture incidence rates

**Figure 3:**
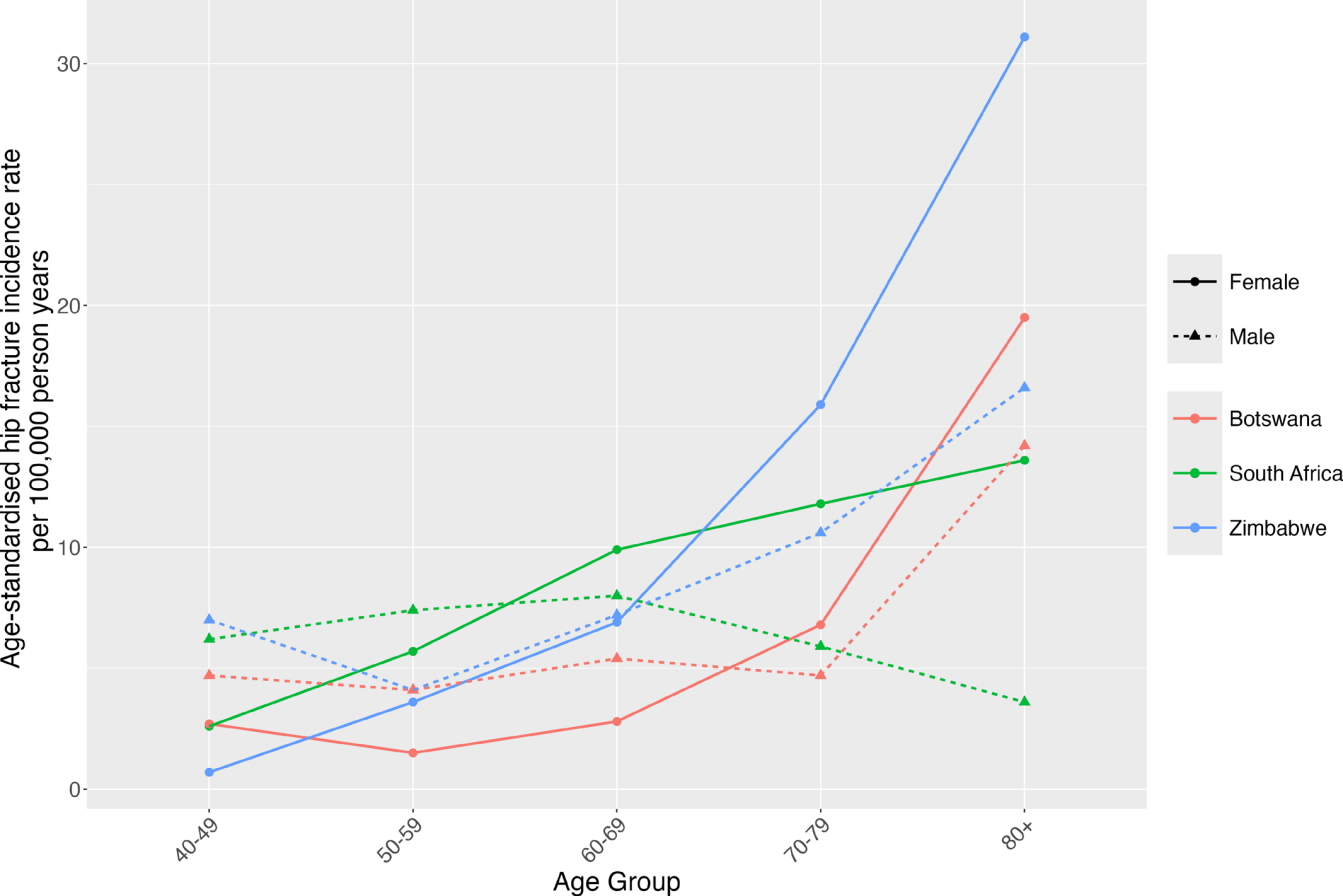
Age-standardised incidence rates for hip fractures in adults aged ≥40 years in Zimbabwe, South Africa and Botswana, stratified by sex.

Age-standardised hip fracture incidence rates are broadly comparable between Zimbabwe, Botswana and Black South Africans in those aged 40–69 years; thereafter, rates in Zimbabwean women and men are higher than those in Botswana and South Africa (figure 3). All countries see higher incidence in men than women under 50 years (7.0 vs 0.7 per 100 000/year in Zimbabwe; 6.2 vs 2.6 per 100 000/year in South Africa (Black population) and 4.7 vs 2.7 per 100 000/year in Botswana, respectively). The highest incidence rates are seen in Zimbabwean women aged ≥80 years (31.1 in Zimbabwe, 13.6 in Black South Africans, 19.5 in Botswana per 100 000 person years).

## Discussion

This is the first prospective study to determine fracture incidence rates in Zimbabwe and compare rates in the region. In both men and women, most hip fractures were fragility fractures consistent with osteoporosis, and thus, a clear association was seen with older age. At young ages, the higher fracture rate seen in men compared with women was largely driven by high-energy fractures, such as road traffic injuries. Between the ages of 50 and 70 years, hip fracture incidence rates were similar between men and women. After 70 years of age, hip fracture incidence increased more in women than in men. At older ages, incidence rates in Zimbabwe exceeded those previously reported in Botswana and Black South Africans.^17 18^ Future projections estimate that hip fracture numbers in Zimbabwe will more than double in both men and women by 2052, putting additional strain on already challenged healthcare systems.

This study identified an unexpectedly high proportion of hip fractures in men, who contributed 45% to event numbers. A systematic review of hip fracture incidence worldwide showed that age-standardised incidence of hip fracture in men was approximately half that in women.^27^ This contrasts to these findings in Zimbabwe, where age-standardised incidence in men was 46.6 and women 58.8 per 100 000. Retrospective case series reports of fragility fractures in Ghana and Tanzania also reported equal sex distributions for hip fracture cases.^28 29^ This may be explained by substantially higher rates of alcohol consumption in men, causing falls and osteoporosis^30 31^ or comparatively lower hip fracture rates in women who are more likely to be obese and thus be protected by padding around the hips.^32^ That 37% of patients took more than 2 weeks to present with arguably one of the most painful injuries of older age, was surprising and in stark contrast to high-income settings where guidelines recommend surgery within 48 hours of a hip fracture injury to improve patient outcomes.^33^,^35^ These delays were not sex- or age-associated, suggesting against prioritisation of younger male health. Reasons for delays may include (i) a lack of awareness of the potential for hip fracture given the low-energy trauma mechanism and osteoporosis as a condition being unknown, (ii) a need to mobilise out-of-pocket finances to access hospital care and/or (iii) a need to find a mode of transport to hospital; qualitative research is needed to better understand such barriers.

While incidence rates in older Zimbabweans exceeded those reported from South Africa and Botswana, incidence rates in these three countries are lower than in other continents. Countries with the highest hip fracture incidence are all high-income countries and include Denmark and Sweden (>400 per 100 000); estimates in Zimbabwe are similar to those in North Africa and Ecuador.^27^ In South Africa, an upper middle-income country, hip fracture incidence is expected to increase over the next 30 years,^24^ while in Zimbabwe, we estimated incidence rates will remain fairly static. This is likely due to different stages of epidemiological transition, where in Zimbabwe (mean life expectancy 59 years),^36^ the largest population increase will be seen in the 40 to 54 age group who are at relatively low fracture risk, for now. However, population ageing is still expected to lead to hip fracture numbers more than doubling in both men and women by 2052. This has significant health policy implications as we have recently shown the healthcare system in Zimbabwe lacks even basic amenities to treat current fracture events.^37^

Higher hip fracture incidence rates in Zimbabwe may reflect higher detection by a prospective study design than the retrospective design employed in Botswana or reflect truly higher incidence due to greater financial hardship, deprivation, malnutrition and lack of basic healthcare following Zimbabwe’s economic downturn; in 2020 Zimbabwe ranked 159 out of 193 countries in the Human Development Index,^38^ and the World Bank estimates 42% of the population lived below the poverty line.^14^

The majority of the hip fractures in this study were fragility fractures indicative of osteoporosis, a musculoskeletal disease characterised by the deterioration of bone architecture, which increases the likelihood of a fragility fracture.^39^ A recent review of the prevalence of osteoporosis in three African countries, including Zimbabwe, highlighted the lack of diagnostic and treatment options for osteoporosis compared to high-income countries.^8^ Low bone mineral density, which contributes to fragility fracture risk, can be measured by dual-energy X-ray absorptiometry (DXA) scanning.^40^ However, only one DXA scanner is available in Zimbabwe for clinical use, and it is housed within a private facility.^37^ As most people access public hospital care, only a small number would likely be able to afford a DXA scan. Alternative tools that consider clinical risk factors beyond bone density are much needed to guide fracture risk assessment and treatment; fortunately, tools such as FRAX^20^ will be able to be calibrated for Zimbabwe using our new data. However, currently no anti-osteoporosis treatment is considered an essential medicine by the WHO.^41^ Our findings demonstrate a growing need to update these global recommendations.

Our study has several strengths: it is the first prospective incidence study of fracture at any skeletal site in Zimbabwe; most hip fractures were confirmed radiographically or by orthopaedic surgeons, despite challenged access to necessary facilities; inclusion of both private and public hospitals maximised generalisability; and the 2022 Zimbabwe Population and Housing Census occurred during our study period (October 2021 to October 2023),^13^ providing contemporaneous population denominators. This 2022 Census also allowed us to compare the actual population numbers to the UN 2022 predicted population and apply a correction factor. The study had limitations. Although a thorough community sensitisation and continuous active case finding for 2 years across all Harare polyclinics minimised the potential for missing community cases, a few hip fracture cases may have been missed. Reasons include those who died shortly after injury and did not present to medical services; one private hospital declined to participate, although numbers at this facility would be expected to be few; 3 months of industrial action in the public sector during the study period may have resulted in some patients seeking treatment elsewhere. The exact population numbers for Harare Province by the sex and age bands were not released in the Census, but only made available for ‘urban areas’, on which our estimates were based; as the capital Harare constitutes the majority of the urban population, this approach was reasonable. The UN data have been shown to underestimate older population numbers in South Africa, as they did here in Zimbabwe.^24^ The correction factor applied to projected UN population estimates is expected to have mitigated differences between UN and census data, but estimates of the population growth remain uncertain given variable death and migration rates. We assumed hip fracture incidence rates in Harare will be generalisable across Zimbabwe; however, the hip fracture risk in rural settings may differ from urban settings, although the evidence is mixed. While more severe injuries in rural settings have been reported in Canada,^42^ a meta-analysis of predominantly high-income countries found that hip fracture incidence was lower in rural areas.^43^ Conversely, in a survey of traumatic fractures in China, no differences between rural and urban incidence were identified.^44^ Our projections only considered potential changes in the population by age and sex, not changes in other potential risk factors such as obesity or HIV prevalence.

In summary, Zimbabwe age-standardised hip fracture incidence rates for men and women aged 40 years and older exceed those previously reported in the region. Most fractures are fragility fractures indicative of osteoporosis, suggesting anti-osteoporosis medicines should be considered essential medicines. Across Zimbabwe, the number of hip fractures occurring annually is expected to increase more than 2.5-fold in the next 30 years, straining an already saturated healthcare system. This study’s data will enable calibration of fracture risk assessment tools, such as FRAX^20^) for routine use in clinical practice in Zimbabwe. Findings can further be used to inform national planning of orthopaedic services.

## supplementary material

10.1136/bmjgh-2024-017365 online supplemental file 1

10.1136/bmjgh-2024-017365 online supplemental file 2

10.1136/bmjgh-2024-017365 online supplemental file 3

10.1136/bmjgh-2024-017365 online supplemental file 4

10.1136/bmjgh-2024-017365 online supplemental file 5

## Data Availability

Data are available upon reasonable request.
