# Supplementary figures and images for: Age- and sex-specific incidence rates and future projections for hip fractures in Zimbabwe
Source: BMJ Glob Health. 2025 Jan 27;10(1):e017365. doi: 10.1136/bmjgh-2024-017365 (PMC11772929; doi:10.1136/bmjgh-2024-017365)

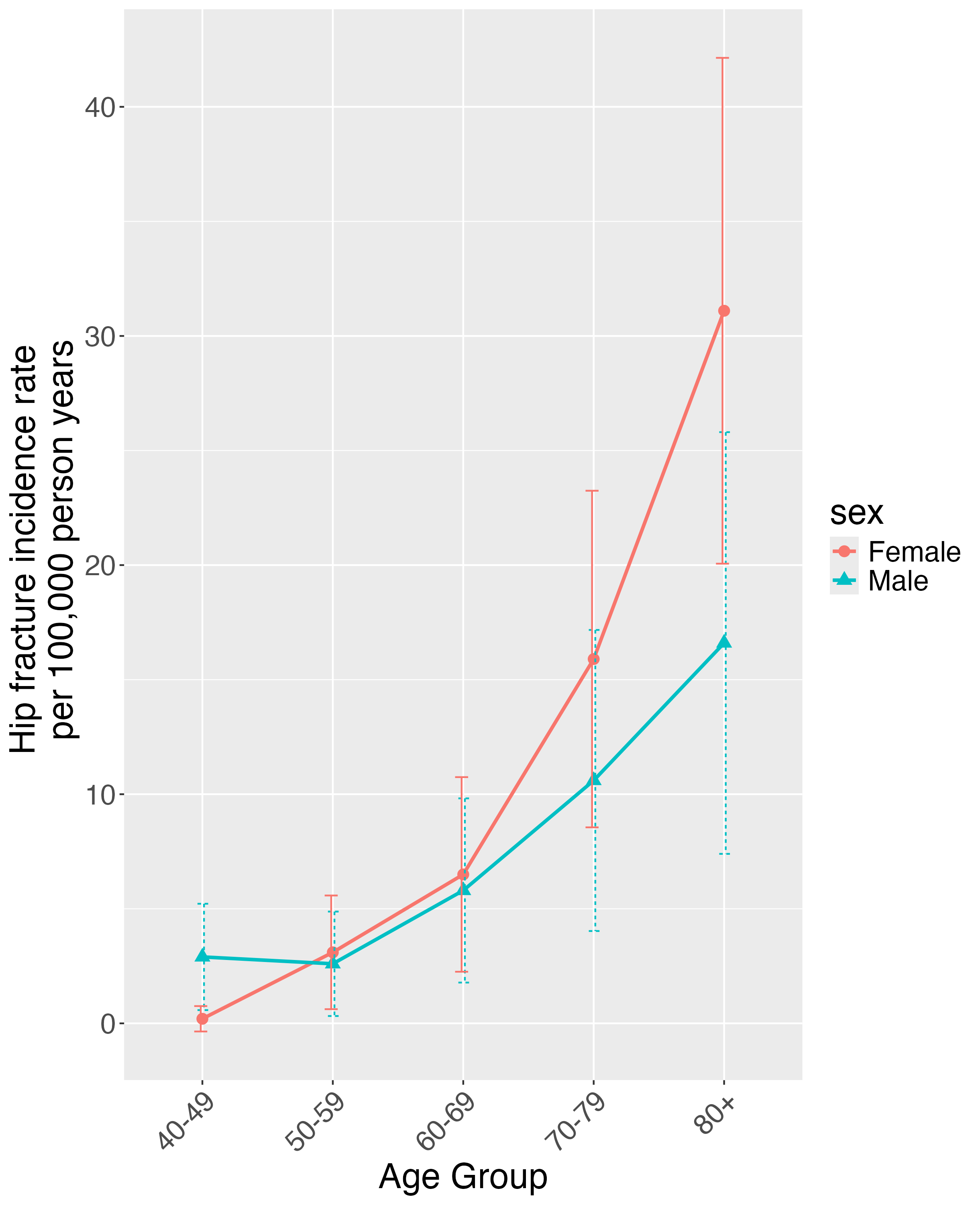

Supplement: online supplemental file 1 [file bmjgh-10-1-s001.tiff]

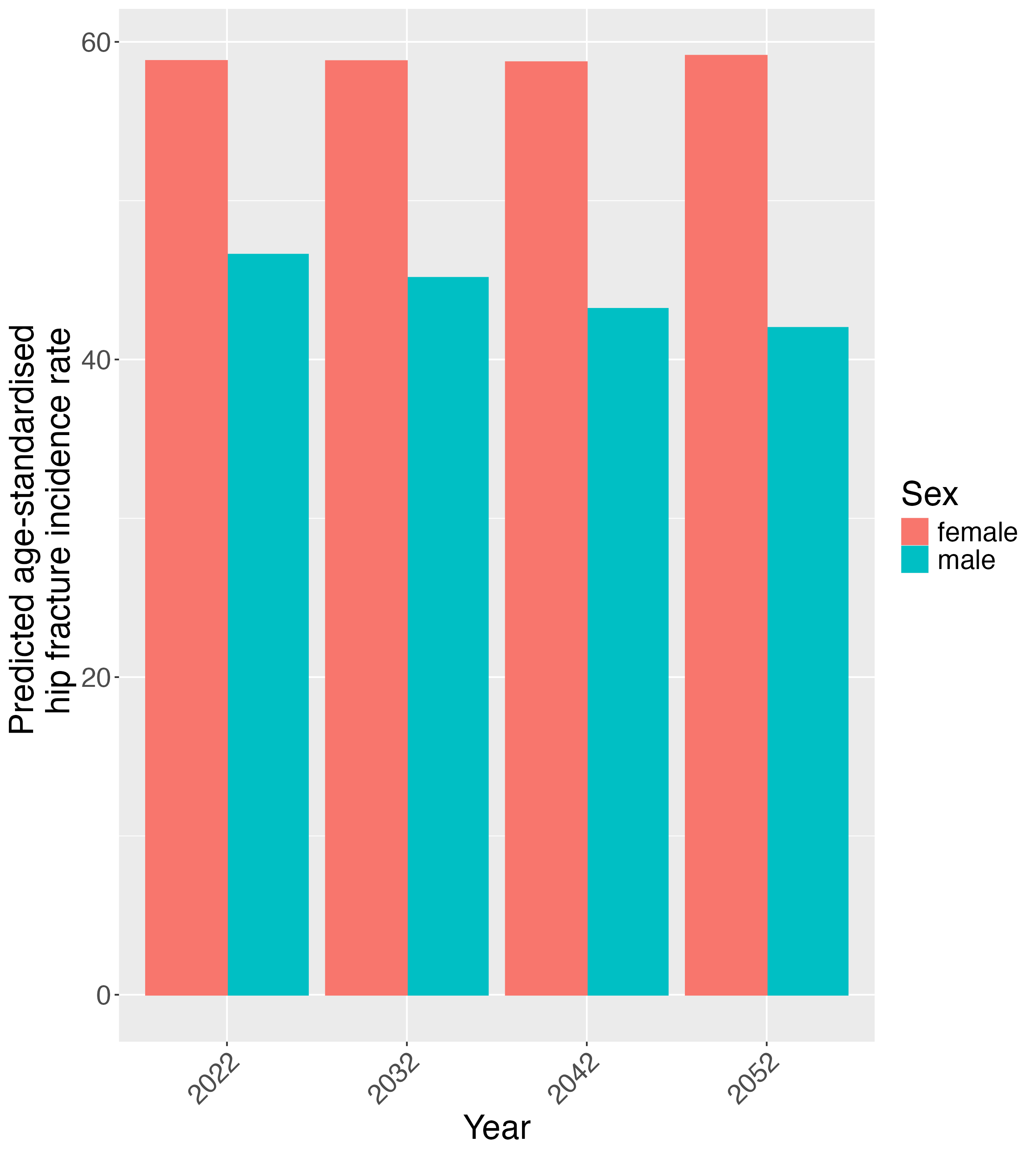

Supplement: online supplemental file 3 [file bmjgh-10-1-s003.tiff]
