# Supplementary material for: Age- and sex-specific incidence rates and future projections for hip fractures in Zimbabwe
Source: BMJ Glob Health. 2025 Jan 27;10(1):e017365. doi: 10.1136/bmjgh-2024-017365 (PMC11772929; doi:10.1136/bmjgh-2024-017365)
Supplement: online supplemental file 4 [file bmjgh-10-1-s004.pdf]

Supplementary Table 1: Hip fracture incidence in five-year age bands, overall and stratified by sex, for Harare Province and Zimbabwe, 2022, excluding high trauma fractures.

Five-year age bands (years)	Number hip fractures[a] per year (n)	Harare Province population (n)	Incidence rate per 100,000 person years	Relative risk ratio (95% CI)	Zimbabwe total population[c] (n)	Incidence rates per 100,000, age-standardised to Zimbabwe population 2022[d]
**Men & Women**						
40-44	4.0	111,012	3.6		795,275	0.9
45-49	2.5	85,744	2.9	0.8 (0.16-3.88)	661,444	0.6
50-54	5.5	57,308	9.6	2.7 (0.74-9.79)	450,604	1.4
55-59	5.5	34,687	15.9	4.4 (1.21-15.95)	309,004	1.5
60-64	7.5	26,881	27.9	7.7 (2.29-25.91)	295,155	2.6
65-69	9.5	20,172	47.1	13.1 (4.07-42.13)	254,317	3.7
70-74	12.5	11,344	110.2	30.6 (9.93-94.33)	171,193	5.9
75-79	15.5	6,848	226.3	62.8 (20.93-188.46)	110,023	7.8
80-84	14.5	4,389	330.4	91.7 (30.33-277.29)	76,105	7.8
85+	28.5	4,140	688.4	191.1 (67.14-543.95)	80,844	17.4
Total over 40	105.5	362,524	29.1	8.1 (2.98-21.98)	3,203,964	49.6
Total over 50	99.0	165,768	59.7	16.6 (6.11-45.1)	1,747,245	48.1
Total over 65	80.5	46,893	171.7	47.6 (17.44-129.91)	692,482	42.6
**Men**						
40-44	4.0	55,727	7.2		385,120	1.9
45-49	2.0	45,200	4.4	0.6 (0.11-3.28)	328,502	1.0
50-54	2.5	30,666	8.2	1.1 (0.23-5.34)	224,187	1.3
55-59	2.5	16,308	15.3	2.1 (0.43-10.2)	128,893	1.4
60-64	3.5	12,063	29.0	4 (0.95-16.79)	117,633	2.3
65-69	4.5	8,878	50.7	7.1 (1.85-27.3)	103,166	3.6
70-74	3.5	4,592	76.2	10.6 (2.53-44.48)	67,666	3.5
75-79	6.5	2,657	244.6	34.1 (9.82-118.42)	44,665	7.5
80-84	3.0	1,680	178.6	24.9 (5.58-111.17)	31,067	3.8

Five-year age bands (years)	Number hip fractures[a] per year (n)	Harare Province population (n)	Incidence rate per 100,000 person years	Relative risk ratio (95% CI)	Zimbabwe total population[c] (n)	Incidence rates per 100,000, age-standardised to Zimbabwe population 2022[d]
85+	9.5	1,441	659.3	91.8 (28.58-294.91)	29,645	13.4
Total over 40	41.5	179,212	23.2	3.2 (1.15-8.93)	1,460,544	39.7
Total over 50	35.5	78,285	45.3	6.3 (2.24-17.71)	746,922	36.8
Total over 65	27.0	19,248	140.3	19.5 (6.82-55.72)	276,209	31.8
**Women**						
40-44	0.0	55,285	0.0		410,155	0.0
45-49	0.5	40,545	1.2		332,942	0.2
50-54	3.0	26,642	11.3	9.1 (0.46-181.67)	226,417	1.5
55-59	3.0	18,379	16.3	13.2 (0.66-263.52)	180,111	1.7
60-64	4.0	14,818	27.0	21.9 (1.16-414.23)	177,522	2.7
65-69	5.0	11,294	44.3	35.9 (1.96-657.08)	151,151	3.8
70-74	9.0	6,751	133.3	108.1 (6.27-1864.51)	103,527	7.9
75-79	9.0	4,191	214.7	174.1 (10.09-3002.7)	65,358	8.0
80-84	11.5	2,709	424.5	344.2 (20.29-5839.68)	45,038	11.0
85+	19.0	2,699	704.0	570.8 (34.44-9460.33)	51,199	20.7
Total over 40	64.0	183,312	34.9	28.3 (1.75-457.38)	1,743,420	57.5
Total over 50	63.5	87,483	72.6	58.9 (3.64-952)	1,000,323	57.3
Total over 65	53.5	27,645	193.5	156.9 (9.69-2541.09)	416,273	51.4
