# Supplementary material for: Age- and sex-specific incidence rates and future projections for hip fractures in Zimbabwe
Source: BMJ Glob Health. 2025 Jan 27;10(1):e017365. doi: 10.1136/bmjgh-2024-017365 (PMC11772929; doi:10.1136/bmjgh-2024-017365)
Supplement: online supplemental file 5 [file bmjgh-10-1-s005.docx]

**REFLEXIVITY STATMENT**

1. **How does this study address local research and policy priorities?**

The Zimbabwean Ministry of Health and Child Care are committed to the principals of Universal Health Coverage, and this research aligns with the goals of the Zimbabwean National Healthy Ageing Strategic Plan 2017-2020.

1. **How were local researchers involved in study design?**

TM, PM, MN, TB, JC and RF were involved in questionnaire design, decisions regarding choice of study sites, community and stakeholder engagement, and securing permissions from hospitals. The team ensured contextual relevance and language translation of materials. The team piloted study tools, processes and questionnaires. TB secured population denominator data from national census information.

1. **How has funding been used to support the local research team?**

Funding has provided secure employment for the team and administrative support staff, a well-equipped office and a dynamic research environment in which to work with regular team meetings; project-related travel expenses have been renumerated.

1. **How are research staff who conducted data collection acknowledged?**

TM, TB and JC, study co-ordinator, data manager, and data assistant respectively, facilitated data collection, cleaning and analysis. TM is joint first author; JC and TS are co-authors. The Zimbabwean research assistants who collected data are all thanked by name in the acknowledgments section of the paper.

1. **Do all members of the research partnership have access to study data?**

All non-anonymised data (consent forms) are held with the team in Zimbabwe and are not accessible to anyone outside of Zimbabwe. Anonymised data are held within a secure REDCap database accessible to both Zimbabwe and United Kingdom team members.

1. **How was data used to develop analytical skills within the partnership?**

Across the wider Fractures-E3 research programme (which includes colleagues in The Gambia and South Africa) we hold monthly Early Career Researcher training seminars for example, including, programming R (utilising data collected in Fractures-E3 projects), Critical Appraisal, Bias and Confounding, Referencing, to name a few. TM was supported and completed a MSc during this project. PM is supported in a MSc which is ongoing.

1. **How have research partners collaborated in interpreting study data?**

We have held monthly, then 6-weekly project management group meetings, plus annual week-long investigator meetings since 2020. Emerging data have been sequentially discussed as a team. HW analysed the data. Co-authors contributed to data interpretation and contextualisation.

1. **How were research partners supported to develop writing skills?**

TM and HW both attended writing courses, TM online from Zimbabwe funded by the NIHR and HW in the UK funded by the University of Bristol. The senior academics on the team supported the early career researchers by giving multiple rounds of feedback on the writing.

1. **How will research products be shared to address local needs?**

These data were presented to Ministry of Health and Child Care, and key stakeholders and health-related NGOs on 19^th^ June 2024, following which a briefing report was sent to all. Findings will be disseminated to the public hospitals from which most data were collected.

1. **How is the leadership, contribution and ownership of this work by LMIC researchers recognised within the authorship?**

TM and HW are lead co-authors on this paper. TM is the study co-ordinator in Zimbabwe and HW is the data manager in the UK. RF and CG are the principal investigators in Zimbabwe and the UK, respectively and are joint last authors. MN is the co-principal investigator in Zimbabwe. Other co-authors include PM (Zimbabwean orthopaedic surgeon), TB and JC (data manager and data assistant Zimbabwe).

1. **How have early career researchers across the partnership been included within the authorship team?**

TM, HW, AB, JC, PM, SH, JM are all early career researchers. TM and HW are lead co-authors. TM, JC, PM are from Zimbabwe and HW, AB, SH and JM are from United Kingdom.

1. **How has gender balance been addressed within the authorship?**

There are eight men (TM, PM, MN, JC, SH, SG, JM and MC) and six women (HW, AB, TB, KW, RF and CG). Three women are lead or last co-authors.

1. **How has the project contributed to training of LMIC researchers?**

TM was supported and completed a MSc during this project. PM is supported in a MSc which is ongoing. An online early career researcher training programme has run since 2021 as part of this research programme (see (6)).

1. **How has the project contributed to improvements in local infrastructure?**

Musculoskeletal research is now a core research theme at The Health Research Unit Zimbabwe; invaluable expertise and field experience have been developed, and the reputation of the Unit has benefited from this investment.

1. **What safeguarding procedures were used to protect local study participants and researchers?**

During project set-up we wrote a project-specific safeguarding policy. Staff training and re-training included safeguarding of staff (e.g. avoidance of lone working in communities, seat belt policy) and of study participants (e.g. intervening on humanitarian grounds when needed). Risk reporting and safeguarding were standing agenda items on 4-6 weekly project management group meetings.
